# Exploring ceramide as a novel biomarker and therapeutic target for Alzheimer’s disease

**DOI:** 10.3389/fnins.2026.1771302

**Published:** 2026-06-05

**Authors:** Wangting Zhao, Xu Liu, Yimin Yao, Ying Yu, Zhengjun Hu

**Affiliations:** The FirstAffiliated Hospital of Zhejiang Chinese Medical University (Zhejiang Provincial Hospital of Chinese Medicine), Hangzhou, China

**Keywords:** AD, ceramide, inflammatory response, insulin resistance, lipid metabolism, mitochondrial homeostasis

## Abstract

Metabolic dysregulation is increasingly being recognized as a hallmark across various neurodegenerative diseases. While Alzheimer’s disease (AD) is well-established as a dual proteinopathy characterized by amyloid-beta (Aβ) deposition and tau protein tangles, the specific mechanisms mediating lipid homeostasis imbalance have garnered increasing attention. However, translating these findings into safe clinical therapeutic targets remains a formidable challenge, primarily hindered by the pleiotropic roles of ceramides in maintaining neural and immune homeostasis, as well as the blood–brain barrier (BBB) penetration issues and systemic safety limitations of current sphingolipid-targeting strategies. We conducted a comprehensive search of electronic databases, including PubMed, Web of Science, and Google Scholar, to identify relevant studies published from database inception through March 2026. The search term combinations included: “Alzheimer’s disease,” “AD,” “ceramide,” “sphingolipid metabolism,” “biomarker,” “therapeutic target,” “neuroinflammation,” and “mitochondrial dysfunction.” To ensure the depth and rigor of this review, priority was given to peer-reviewed original research, systematic reviews, and meta-analyses. The search was restricted to English-language literature. Additionally, the reference lists of retrieved articles were manually screened to identify further relevant studies. This narrative review aims to comprehensively elucidate the potential roles of ceramides in AD pathogenesis, exploring their associations with triggering inflammatory responses, mediating apoptosis, interfering with signal transduction, and inducing mitochondrial dysfunction.

## 1 Introduction and significance

AD is the most prevalent neurodegenerative disorder, with its classic pathological hallmarks characterized by Aβ deposition, neurofibrillary tangles (NFTs), and neuroinflammation (Guerreiro et al., 2012; Holtzman et al., 2012; Serrano-Pozo et al., 2021). Despite an increasing understanding of its risk factors, the underlying pathogenesis remains incompletely understood. Consequently, the role of sphingolipid metabolism and its core component, ceramide, in age-related metabolic alterations has garnered growing attention (Hussain et al., 2020; Baloni et al., 2022). Numerous studies have shown that elevated ceramide levels modulate secretase activity, thereby accelerating the *β*- and *γ*-cleavage of amyloid precursor protein (APP) and leading to subsequent Aβ deposition (Cordy et al., 2003; Kalvodova et al., 2005). Specific species, such as Cer16:0 and Cer18:0, not only serve as disease risk indicators but also further perturb the cerebral immune environment by regulating peripheral T-cell differentiation (Sun et al., 2025; Mielke et al., 2012). These findings suggest that ceramides could potentially serve as early diagnostic biomarkers and therapeutic targets for AD. Future research is warranted to further elucidate the specific mechanisms of ceramides in AD and to explore how the modulation of ceramide metabolism can facilitate the development of novel therapeutic strategies aimed at decelerating AD progression and improving cognitive function in patients.

## 2 Biological properties of ceramide and lipid metabolic dysregulation

The brain is one of the most lipid-dense organs, rich in myelin, with lipids constituting approximately 50–60% of its dry weight. These lipid components participate in cellular transport, signaling pathways, energy storage, neurogenesis, and the formation of membranes and myelin (Michikawa, 2006). Ceramide synthesis primarily occurs through three types of pathways: the *de novo* synthesis pathway, the sphingomyelin hydrolysis pathway, and the salvage pathway. As core biomolecules, ceramides exert functional regulatory roles through these various synthetic routes (Hannun and Obeid, 2011; Mencarelli and Martinez-Martinez, 2013; Clarke et al., 2006). Key enzymes in both the de novo and salvage pathways, known as ceramide synthases (CerS), comprise six isoforms (CerS1–CerS6), five of which are expressed in the brain. Each CerS exhibits a preference for specific fatty acyl-CoA substrates, thereby generating particular ceramide species with unique N-linked fatty acids. These ceramide species vary in chain length (C14–C26), are distributed across different cellular domains, and likely execute specific functions (Hannun and Obeid, 2011; Mencarelli and Martinez-Martinez, 2013). Through these various chain-length modifications, they perform regional neuronal maintenance functions (Lyn-Cook et al., 2009; Diaz-Vegas et al., 2023). A comprehensive lipidomics atlas of the nervous system has further elucidated the biological significance of this chain-length specificity: namely, that oligodendrocytes—the primary myelinating cells of the central nervous system—synthesize an abundance of very-long-chain (VLC) ceramides (such as C24:0 and C24:1). These VLC species are essential for maintaining myelin structural integrity and ensuring efficient long-range axonal conduction; in contrast, shorter-chain species (such as C16:0 and C18:0) are more enriched in other neuronal and glial domains, where they exert unique signaling regulatory functions (Wang et al., 2021).

However, abnormalities such as systemic issues induced by a chronic high-fat diet (HFD) can significantly augment synthetic pathways, triggering the accumulation of abnormal ceramide pools in the brain (Kogot-Levin and Saada, 2014). Persistent accumulation leads to mitochondrial respiratory chain dysfunction, which adversely affects fatty acid *β*-oxidation and results in lipid metabolic disorders (Fisher-Wellman et al., 2021; Mecocci et al., 1996). Previous studies have indicated that abnormal changes in ceramide metabolism may occur in AD patients. In a study by Mielke et al. (2012), it was found that, compared to control populations, elevated serum ceramide levels were significantly associated with an increased risk of Alzheimer’s disease, suggesting that an imbalance in ceramide homeostasis may participate in the onset and progression of AD.

Dysregulation of the lipid metabolic network may not only reflect changes in the overall metabolic status of the body but also interfere with key biological processes—such as cell membrane structural stability, signal transduction, and the regulation of inflammatory responses—by affecting the balance of ceramide synthesis, transport, and degradation. Based on this, ceramides and their related lipid metabolic pathways may play a pivotal bridging role between lipid metabolic abnormalities and the pathological progression of AD, potentially serving as important leads for early disease identification and potential intervention.

## 3 Pathogenic mechanism branches of ceramide in AD progression

### 3.1 Amplification of pro-inflammatory cytokine release

**Figure 1:**
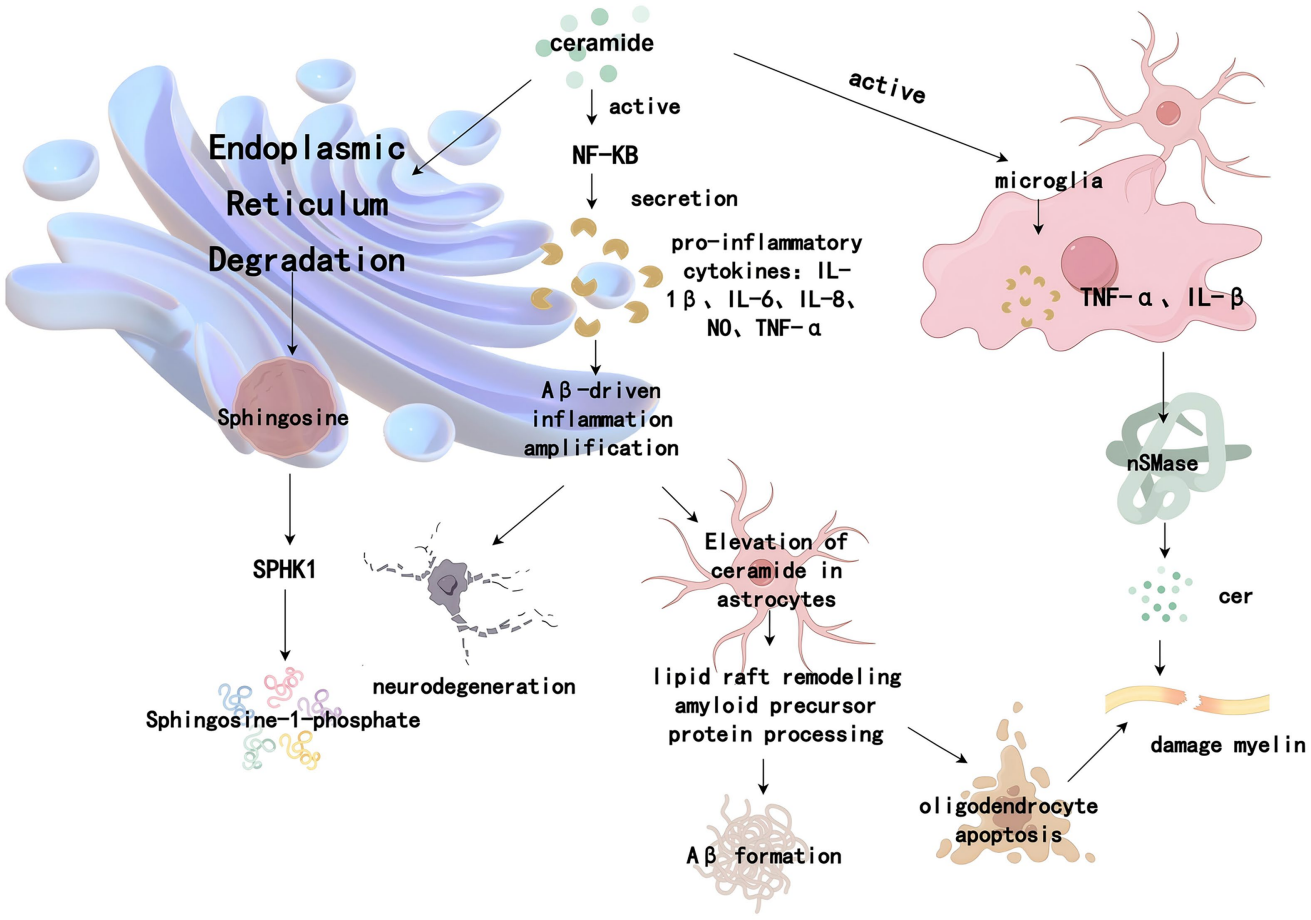
Ceramide triggers glia-mediated inflammatory cytokine release and activates neutral sphingomyelinase to amplify Aβ toxicity, culminating in neuronal degeneration.

Ceramide, as a core molecule of sphingolipid metabolism, plays a pivotal role in driving and amplifying neuroinflammation in AD and other neurodegenerative diseases. This involvement is highly cell-type specific: in astrocytes and microglia, C16:0 and C18:0 are the two most significantly elevated pathogenic subspecies, which are closely linked to pro-inflammatory signaling; notably, in APOE4 carriers, hyperactivation of astrocytes is highly correlated with this abnormal accumulation (den Hoedt et al., 2021). In contrast, oligodendrocytes exhibit a distinct sphingolipid profile dominated by very-long-chain (VLC) ceramides (e.g., C24:0, C24:1), which are essential for myelin structural integrity rather than simple inflammatory mediation. Recent evidence from microglia-depletion studies further underscores this cellular heterogeneity, highlighting microglia-specific regulation of lipid metabolism as a critical factor in AD pathology (Xu et al., 2025). Mechanistically, TNF-*α*-induced sphingomyelinase (SMase) activity promotes the large-scale production of long-chain ceramides in astrocytes and microglia. Excessive Cer16:0 and Cer18:0 not only serve as intracellular second messengers but also strongly drive a feed-forward inflammatory cascade by activating the transcription factor NF-κB (Holtzman et al., 2012). This induces the robust expression of TNF-α, IL-1β, IL-6, IL-8, monocyte chemoattractant protein-1 (MCP-1), and nitric oxide (NO), while upregulating the activities of pro-inflammatory enzymes such as cyclooxygenase-2 (COX-2) and lipoxygenase (LOX) (Schütze et al., 1992; Woida and Satchell, 2022; Lukiw and Bazan, 1998). Notably, the triggering mechanisms of such pro-inflammatory signaling are expanding beyond intracerebral stress toward systemic metabolic crosstalk. Latest evidence indicates that bacterial extracellular vesicles (bEVs) derived from gut microbiota can bridge the gut-brain axis, significantly inducing microglial polarization toward a pro-inflammatory phenotype and accelerating Aβ deposition. This suggests that gut-derived external stimuli may serve as a primary initial stressor for the aforementioned ceramide-dependent inflammatory burst (Xie et al., 2025). This ceramide-dependent amplification of inflammatory responses is universal across various brain pathologies; for instance, TNF-*α* relies on this pathway to impair the viability of hypothalamic GT1-7 neurons (Sortino et al., 1999). The abnormal accumulation of d18:1/16:0 ceramide in the brains of patients with Pick’s disease has also been confirmed to correlate with astrogliosis, suggesting that elevated ceramide is likely a common pathological hallmark of neuroinflammation in neurodegenerative disorders (de Wit et al., 2019). Furthermore, sphingosine produced by ceramide degradation in the endoplasmic reticulum (and subsequently converted to sphingosine-1-phosphate via SPHK1) is also a pro-inflammatory mediator, which, together with the parent molecule, maintains the inflammatory feed-forward loop. During this period, persistently elevated ceramide also induces cell membrane lipid remodeling in glial cells and neurons, thereby promoting abnormal APP processing and the formation of Aβ plaques (Dehghan et al., 2022). Given that ceramide occupies a central hub position in complex neuroinflammatory networks, it has become a key intervention node for modulating the AD inflammatory microenvironment. Experiments have conversely confirmed that intervening to reduce ceramide levels in AD models can effectively inhibit the polarization of microglia toward a pro-inflammatory phenotype, thereby significantly alleviating the neuroinflammatory burden under pathological conditions (Crivelli et al., 2021). Synthesizing the evidence presented above, ceramide serves not merely as a byproduct of lipid metabolic dysfunction in AD but as a pivotal hub orchestrating the neuroinflammatory cascade. To provide a holistic view of this multidimensional interaction network, we have integrated these pathways into a model of ceramide-mediated pro-inflammatory exacerbation (Figure 1). This model delineates the ‘priming effect’ of ceramide on pro-inflammatory cytokine production via the NF-κB axis, as well as the ‘inflammatory amplification loop’ driven by Aβ within microglia. This cycle not only promotes astrocytic Aβ deposition but also induces oligodendrocyte apoptosis and myelin impairment through the activation of neutral sphingomyelinase (nSMase). Such intricate cross-cell and multi-pathway feedback systems elucidate why AD-associated neuroinflammation remains recalcitrant to single-target interventions.

### 3.2 Mediator of cellular apoptosis

Ceramides trigger apoptosis through multiple mechanisms, including mitochondrial depolarization, reactive oxygen species (ROS) generation, cytochrome c release, Bcl-2 depletion, and caspase-3 activation. Aβ, a cleavage product consisting of 39–43 amino acids derived from the amyloid precursor protein APP, is considered a primary neurotoxic factor in the pathogenesis of AD and is closely associated with neuronal degeneration (Cutler et al., 2004). In preclinical studies, toxicity triggered by Aβ enhances TNF-mediated iNOS expression. In rigorous experiments focusing on oligodendrocytes (OLGs) (e.g., the work of Lee et al.), Aβ and TNF jointly activated neutral sphingomyelinase (nSMase), leading to severe intracellular oxidative stress and glutathione (GSH) depletion, ultimately driving OLGs toward irreversible apoptosis (Zeng et al., 2005; Lee et al., 2004; Jana and Pahan, 2004). This mechanism is further supported by longitudinal data from AD transgenic models; for instance, in APP(SL)/PS1 knock-in mice, cortical ceramide levels rise significantly at an early stage, which either precedes or coincides with the onset of overt neuronal loss (Barrier et al., 2010). These findings position ceramide as a critical mediator in the execution of the neuronal death program initiated by oxidative stress and Aβ toxicity (Jazvinšćak Jembrek et al., 2015). Research in aging fibroblasts has similarly found that ceramide accumulation can trigger DNA double-strand breaks, activating subsequent senescence arrest and death (Mouton and Venable, 2000; Peacocke and Campisi, 1991). Mendelian randomization analysis has also revealed the central role of specific subspecies (e.g., d42:2 ceramide) in mediating this degenerative transition (Thadathil et al., 2021; Hu et al., 2025; Smale et al., 1995). Post-mortem pathology has definitively confirmed these findings: in the brain tissues of late-stage AD patients, high expression of poly(ADP-ribose) polymerase-1 (PARP-1) and extremely high rates of apoptosis align closely with the predictions of the aforementioned molecular pathways (Cutler et al., 2004; Anderson et al., 2000; Engidawork et al., 2001).

### 3.3 Interference with intra-network signal transduction

Excessive substrates can directly take over key signaling proteins. Abnormally elevated ceramides specifically activate death-associated protein kinase 1 (DAPK1), causing it to phosphorylate the Ser350 site of N-myc downstream-regulated gene 2 (NDRG2), thereby initiating and amplifying the caspase-dependent apoptotic pathway (You et al., 2017; Chalfant et al., 1999). In DAPK1-knockout mice, this pattern of rapid neuronal death is significantly inhibited (Lee et al., 2000). Furthermore, rather than directly targeting the phosphatase itself, long-chain ceramides activate protein phosphatase 2A (PP2A) by directly binding to its endogenous inhibitor, SET/I2PP2A, thereby relieving its inhibitory constraint on the enzyme (Mukhopadhyay et al., 2009). This aberrant activation of PP2A not only suppresses cyclin-dependent kinase 2 (CDK2)—leading to a catastrophic arrest of the normal neuronal cell cycle (Jazvinšćak Jembrek et al., 2015; de Mello et al., 2009; Wang et al., 2020)—but more importantly, in metabolic and neurodegenerative contexts, it strongly dephosphorylates and inactivates the Akt (Protein Kinase B) survival signaling pathway. The dismantling of these crucial neuroprotective networks profoundly accelerates neuronal apoptosis driven by ceramide dyshomeostasis.

### 3.4 Synergistic interaction between T2DM and systemic organs

**Figure 2:**
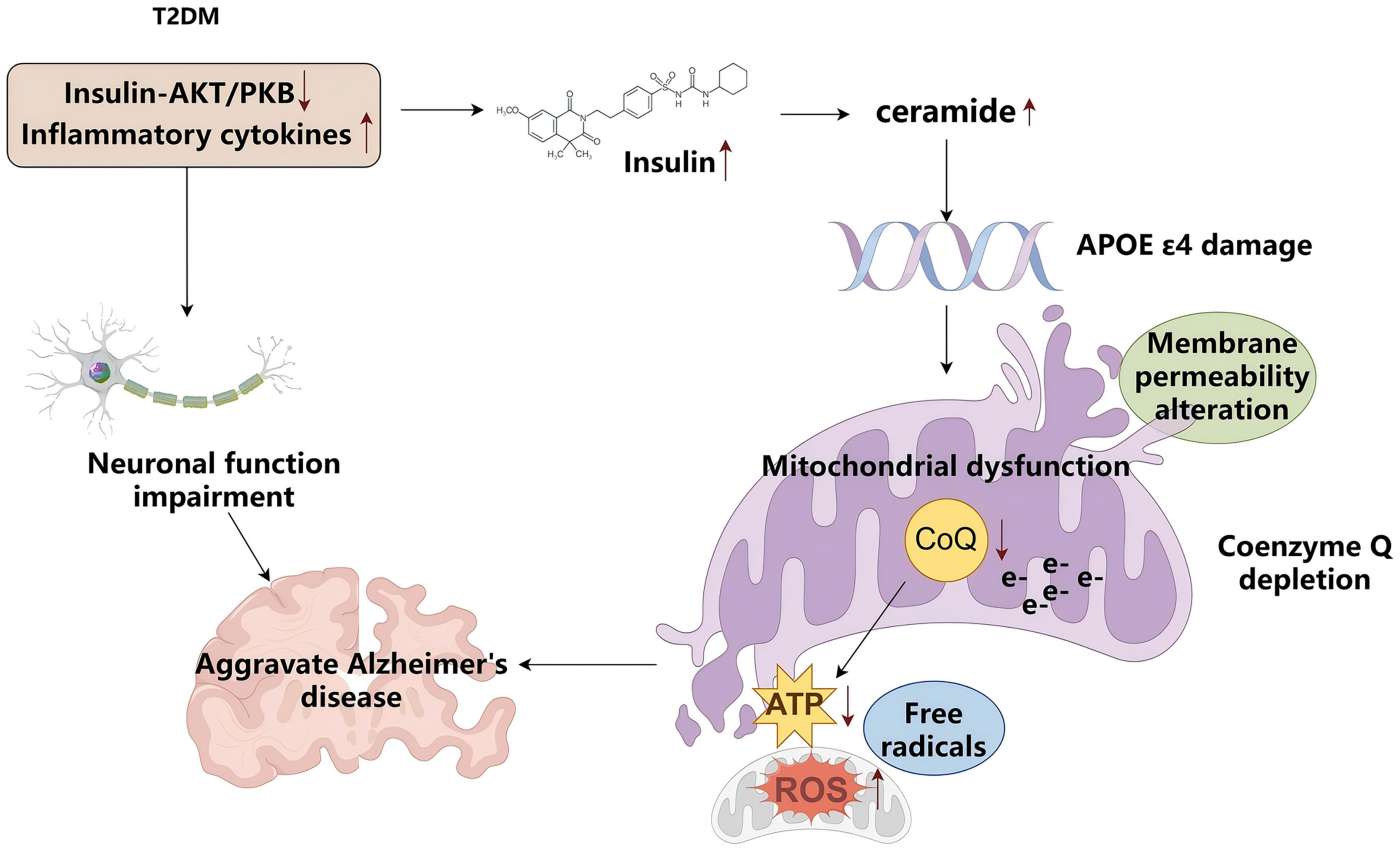
Ceramide impairs mitochondrial function in type 2 diabetes by generating free radicals, disrupting the electron transport chain, and increasing mitochondrial membrane permeability.

Systemic metabolic diseases involving organ crosstalk, such as T2DM and obesity, are critical comorbid foundations for AD. Elevated levels of circulating ceramide in patients are regarded as the primary trigger for severe brain insulin resistance (de Mello et al., 2009; Carr et al., 2023). This pathology is accompanied by a severe deficiency of targeted kinases and mitochondrial degradation within the skeletal muscle framework (Wang et al., 2020). Crucially, research has shown that obesity-induced diabetes triggers the early disruption of nerve mitochondrial and myelin lipid homeostasis, suggesting that lipid dysregulation is a primary pathological event linking metabolic stress to nerve damage (Palavicini et al., 2020). In structural assays of myocytes and brain mitochondria, excess ceramide not only disrupts the electron transport chain (ETC) but also significantly reduces coenzyme Q (CoQ) content and alters membrane permeability (Norat et al., 2020; Takahashi et al., 2017). Following insulin infusion (to induce hyperinsulinemia), brain Cer16:0, Cer20:0, and Cer24:1 are elevated in mice carrying the APOE4 allele, which correlates with impaired cerebral mitochondrial respiration and reduced oxygen consumption (Carr et al., 2023). Furthermore, toxic ceramides synthesized abnormally in the periphery (liver) can readily penetrate the blood–brain barrier (BBB), leading directly to a rapid surge of 4-hydroxy-2-nonenal (HNE) peroxides within the brain (Cordy et al., 2003; Lyn-Cook et al., 2009; Dowlati et al., 2010). Additionally, systemically elevated inflammatory profiles are considered to have profound pathological correlations with neuropsychiatric degeneration, such as depression and severe anxiety disorders (Gulbins et al., 2015; Laurén et al., 2009). To summarize the above research, the pathological link between T2DM and AD is not only reflected in classic insulin resistance and inflammatory responses, but also in the metabolic pathway remodeling induced by both. To systematically elaborate on this metabolic-pathological axis, this article summarizes an integrated model of T2DM aggravating the progression of AD (Figure 2). The model emphasizes how ceramide, after accumulating along with the T2DM disease course, produces a ‘synergistic amplification effect’ with APOE ε4 damage to attack neuronal mitochondria. This attack leads to mitochondrial membrane homeostasis imbalance and Coenzyme Q depletion, causing neurons to fall into an ‘energy-oxidation’ dual crisis of insufficient ATP supply and excessive ROS accumulation. This mechanism not only explains the molecular basis for the increased AD risk in T2DM patients, but also provides a theoretical basis for intervening in metabolic-related AD by targeting mitochondrial function.

### 3.5 Induction of irreversible oxidative stress and mitochondrial dysfunction

From a broader pathological perspective, mitochondrial dysfunction may serve as the common hub connecting peripheral metabolic stress and central nervous system injury. Reviews have highlighted that mitochondrial diseases not only trigger energy metabolism failure but also manifest as cognitive impairment, suggesting that brain tissue is highly sensitive to disturbances in mitochondrial homeostasis (Finsterer, 2012). Furthermore, research on ischemic brain injury underscores the critical role of mitochondria in neuronal survival, redox balance, and cellular stress responses, indicating that the nervous system is more vulnerable to metabolic and inflammatory insults when mitochondrial function is compromised (Liu et al., 2018). Therefore, although these studies do not specifically target the ceramide pathway in AD, they provide robust support for the “peripheral metabolic abnormality–mitochondrial imbalance–neurodegenerative change” pathological axis (Norat et al., 2020; Finsterer, 2012).

As an early prodromal phenomenon of AD, oxidative stress is co-catalyzed by Aβ and the dysregulated accumulation of lipid pools (Yasumoto et al., 2019). Gene Set Enrichment Analysis (GSEA) has demonstrated the downregulation of mitochondrial biochemical input pathways and oxidative phosphorylation (OXPHOS) in patients (Shen et al., 2019; Fukuyama et al., 1994). Experiments on purified myelin from mouse brains found that TNF release activates myelin-associated acid sphingomyelinase (aSMase), generating ceramide that potentially compromises myelin integrity (Chakraborty et al., 1997). Additionally, myelin isolation experiments proved that ceramide drives the accumulation of (ROS) via the intense activation of NADPH oxidase, leading to the obstruction of adenosine triphosphate (ATP) production and the total collapse of the calcium buffering system (Rosen et al., 2010; Caspersen et al., 2005; Kolberg et al., 2023). The damage resulting from prolonged local oxidation deprives neural networks of essential membrane conduction potentials, creating a fertile ground for degeneration (Dehghan et al., 2022). Beyond damaging mitochondria, ceramide-triggered ROS accumulation synergistically induces lipid peroxidation. Notably, recent research indicates that lipid metabolic disorders in AD often activate ferroptosis, a pathway potentially reversible through the modulation of specific lipids such as LPC (Zha et al., 2025). Consequently, it appears that ceramide-mediated apoptosis and lipid imbalance-driven ferroptosis synergistically form the physiological basis for neuronal demise in AD (Zha et al., 2025). Notably, another study indicated that myriocin, an inhibitor of *de novo* ceramide biosynthesis, can reverse ceramide accumulation and mitochondrial dysfunction in the brains of animal models (Carr et al., 2023).

### 3.6 Structural support for pathological protein aggregation

Compared to age-matched controls, ceramide levels in AD brains exhibit an explosive growth, increasing as much as three-fold (Han et al., 2002). Studies in Chinese Hamster Ovary cells demonstrate that the non-physiological, short-chain analog C6-ceramide not only upregulates amyloid protein levels but also serves as a biochemical anchor, significantly enhancing the structural stability of *β*-secretase 1 (BACE1) (Dakterzada et al., 2024; Kim et al., 2017). More critically, extracellular vesicles (EVs) enriched with synthesized ceramides have been identified as the “core express carriers” mediating the inter-neuronal propagation of abnormally free phosphorylated tau between the hippocampus and the cortex (Tallon et al., 2023; Asai et al., 2015; Dinkins et al., 2016). Pharmacological blockade of neutral sphingomyelinase 2 (nSMase2) formation effectively reduces the microscopic dissemination area of tau (Mielke et al., 2010; Mielke et al., 2017; Saunders et al., 1993). Furthermore, retrospective measurements of cerebrospinal fluid (CSF) profiles—comprising a 58-month longitudinal follow-up of 91 AD patients and 92 individuals with mild cognitive impairment (MCI) using LC-ESI-QTOF-MS/MS—revealed that CSF C26:0 and Cer(d38:4) are strongly correlated with Aβ42 abnormalities, demonstrating substantial potential for auxiliary molecular diagnosis (Ossenkoppele et al., 2015; Yang et al., 2023; Bandaru et al., 2009). It is important to note that the ceramide profile in AD is highly heterogeneous. While widespread elevations are reported, these findings are often context-dependent, varying with disease stage, brain region, and genetic background. Furthermore, the question of whether ceramide accumulation is a proactive driver or a reactive byproduct of neurodegeneration remains open, cautioning against a simplistic interpretation of the ceramide pathogenicity model.

### 3.7 Genomics-based regional heterogeneity associated with APOE ε4

Based on multi-cohort genetics, populations carrying the core genetic risk locus APOE ε4 exhibit abnormally high expression of Cer(d18:1/24:0) in brain tissues, specifically within the cortical regions (den Hoedt et al., 2021; Couttas et al., 2018; Sienski et al., 2021). Comparisons of neuroanatomical structures further indicate that the hippocampus and cortex possess distinctly different tolerance levels toward ceramide accumulation triggered by this genetic mutation, suggesting that the destructive impact of this molecular target exhibits significant regional preference (Gao et al., 2020).

### 3.8 High-resolution spatial mass spectrometry imaging

Contemporary cutting-edge Matrix-Assisted Laser Desorption/Ionization Mass Spectrometry Imaging (MALDI-MSI) probes have revealed more pronounced features of pathogenic involvement for this molecular class (Ossenkoppele et al., 2015; Yang et al., 2023; Kaya et al., 2017; Michno et al., 2024). Spatial imaging further demonstrates that ceramide and related lipids are not uniformly distributed; instead, they are distinctly plaque-enriched, exhibiting significant spatial co-localization with Aβ plaques and their immediate microenvironment (including the plaque cores), suggesting their potential involvement in the formation and diffusion of the local pathological microenvironment (Kaya et al., 2017; Michno et al., 2024). Imaging-based anatomy clearly indicates that their spatial deposition network is not in a physiological free state but is precisely anchored to and overlapping with the plaque cores and their associated peripheral halos where damage is most severe. At the direct micro-anatomical level, this places specific lipids within the sequence of “pathological matrix accomplices.” However, these conclusions rely heavily on experimental constructs and transgenic rodent verification; in terms of human observation, a substantial proportion are merely cross-sectional correlations. Within the complex inflammatory compensatory surges of the aging, degenerating brain, certain ceramide spikes may also partially reflect “results”—such as passive lipid remodeling or reactive attenuation—requiring maintained objective restraint regarding their role as independent drivers.

## 4 Clinical progress and evaluation of independent calibration

With the continuous accumulation of evidence from large-scale genetics and fluid biomarkers, genetic variants related to lipid transport, such as ABCA7, have been proven to be closely associated with AD risk and clinical heterogeneity, suggesting that lipid metabolism abnormalities may play a significant role in disease stratification (Flury et al., 2025). Building on this, Baloni et al. further pointed out through integrated multi-omic analyses that the ceramide/sphingomyelin (Cer/SM) pathway exhibits a systemic imbalance in AD. This alteration can be detected in both brain tissue and peripheral samples, suggesting that the pathway not only reflects disease-related metabolic reprogramming but may also participate in the key processes of disease onset and progression (Baloni et al., 2022). At the same time, recent longitudinal medical cohort studies have shown that dynamic changes in specific acyl-chain ceramides provide an early prediction window for identifying the progression from MCI to dementia, further supporting their potential as prognostic biomarkers (Kim et al., 2017; Mielke et al., 2010; Mielke et al., 2017; Saunders et al., 1993; Flury et al., 2025). Moreover, under the monitoring of CSF profiles, combinations of multiple subtypes have even demonstrated the potential to substitute for traditional monitoring of apolipoproteins and lesion sites during certain periods (Saunders et al., 1993; Ossenkoppele et al., 2015; Yang et al., 2023; Bandaru et al., 2009; Couttas et al., 2018; Sienski et al., 2021).

## 5 Current limitations in pharmacological evaluation of targets

Although intervening in the ceramide metabolic pathway is biologically rational in theory and has demonstrated certain disease-modifying potential, its translation into clinical treatment still faces significant obstacles. First, gene network regulation strategies centered on ceramide transfer protein long isoform (CERTL) suggest that it can reduce C16-associated lipid accumulation and attenuate specific microglial toxic responses in 5xFAD animal models (Crivelli et al., 2021). However, such intervention methods based on exogenous proteins or molecular reprogramming still face clear limitations regarding BBB penetration, maintenance of long-term stable expression, and avoidance of off-target effects; thus, their clinical application value requires further validation. Second, FTY720, a modulator functionally related to sphingosine-1-phosphate (S1P), has been shown to reduce cerebral ceramide load and improve certain neuropathological phenotypes in E4FAD pathological models (a familial AD hybrid model expressing human APOE4) (Wu et al., 2024; Crivelli et al., 2022). Nevertheless, it is noteworthy that the systemic application of such drugs may be accompanied by adverse reactions such as bradycardia and immunosuppression, making safety a key issue limiting further development, especially for elderly patients with multiple underlying diseases. Finally, inhibition strategies targeting nSMase2 and the class of Functional Inhibitors of Acid Sphingomyelinase (FIASMAs) have shown potential in animal models to delay tau propagation and reduce ceramide-related pathological changes (Tallon et al., 2023; Chowdhury et al., 2022; Kornhuber et al., 2010). However, these drugs typically affect lysosomal function, membrane lipid homeostasis, and various basic physiological processes simultaneously. Therefore, while enhancing intracerebral accessibility, how to avoid disrupting normal synaptic function and triggering extensive anticholinergic-like side effects remains a major challenge that must be addressed during clinical translation. Furthermore, non-specific drugs such as ibuprofen also reshape the cerebral sphingolipid profile, suggesting inherent risks in lipid metabolism intervention (Radermacher et al., 2025). Furthermore, to address the challenge of high invasiveness in current AD delivery systems, latest research has leveraged iPSC technology to develop ‘tentacled’ extracellular vesicles (TenSCev) with innate neural-targeting capabilities. When integrated with bioluminescent optogenetics, this platform achieves functional neuronal remodeling and the clearance of pathological deposits without the need for invasive optical fiber implantation. This precision modulation strategy, based on engineered vesicles, holds the potential to provide critical technical support for overcoming the safety and targeting bottlenecks of ceramide-related targets in clinical translation (Zhai et al., 2025). Overall, while ceramide-related targets provide a noteworthy direction for intervention, a significant translational distance remains between preclinical efficacy and acceptable clinical benefit.

## 6 Conclusion

In summary, this narrative review systematically elucidates the multidimensional roles of ceramides within the complex pathological network of AD. It underscores their significance as bioactive mediators associated with driving neuroinflammation, mediating apoptosis, interfering with signal transduction, inducing mitochondrial dysfunction, and modulating the propagation of pathological proteins. Recent advances in high-resolution spatial mass spectrometry imaging have revealed the precise deposition of ceramides surrounding Aβ plaques, further strengthening the evidence chain for their involvement in the deterioration of the local pathological microenvironment. These findings suggest that the homeostatic imbalance of ceramides may serve as a critical nexus linking systemic metabolic stress to central neurodegeneration.

However, substantial translational barriers remain before ceramide modulation can become a viable therapy. First, achieving pharmacological selectivity without inducing detrimental off-target effects remains highly difficult given the constitutive roles of sphingolipids in basal cellular function. Second, drug delivery systems must overcome blood–brain barrier (BBB) restrictions to achieve efficacious central concentrations. Third, current evidence heavily relies on preclinical models or human cross-sectional associations; therefore, large-scale, longitudinal human trial data are urgently needed. Ultimately, bridging these gaps is essential to clearly define the risk–benefit profile of ceramide-targeted interventions.

In conclusion, while the ceramide pathway offers a highly potential window for early risk stratification and precision intervention in AD, establishing it as a safe and effective clinical target requires vigilant avoidance of single-factor mechanistic attribution, alongside more rigorous causal validation and the exploration of multi-target synergistic strategies.

## Glossary

AD: Alzheimer’s disease
Aβ: Amyloid-beta
NFTs: Neurofibrillary tangles
APP: Amyloid precursor protein
CerS: Ceramide synthases
HFD: High-fat diet
SMase: Sphingomyelinase
MCP-1: Monocyte chemoattractant protein-1
NO: Nitric oxide
COX-2: Cyclooxygenase-2
LOX: Lipoxygenase
SPHK1: Sphingosine-1-phosphate
ROS: Reactive oxygen species
OLG: Oligodendrocyte
nSMase: Neutral sphingomyelinase
GSH: Glutathione
PARP-1: Poly(ADP-ribose) polymerase-1
DAPK1: Death-associated protein kinase 1
NDRG2: N-myc downstream-regulated gene 2
PP2A: Protein phosphatase 2A
CDK2: Cyclin-dependent kinase 2
ETC: Electron transport chain
CoQ: Coenzyme Q
BBB: Blood–brain barrier
HNE: 4-hydroxy-2-nonenal
GSEA: Gene Set Enrichment Analysis
OXPHOS: Oxidative phosphorylation
aSMase: Acid sphingomyelinase
ATP: Adenosine triphosphate
BACE1: β-secretase 1
EV: Extracellular vesicle
nSMase2: Neutral sphingomyelinase 2
CSF: Cerebrospinal fluid
MCI: Mild cognitive impairment
MALDI-MSI: Matrix-Assisted Laser Desorption/Ionization Mass Spectrometry Imaging
SM: Sphingomyelin
CERTL: Ceramide transfer protein long isoform
S1P: Sphingosine-1-phosphate
FIASMAs: Functional Inhibitors of Acid Sphingomyelinase
